# A Lightweight Person Detector for Surveillance Footage Based on YOLOv8n

**DOI:** 10.3390/s25020436

**Published:** 2025-01-13

**Authors:** Qicheng Wang, Guoqiang Feng, Zongzhe Li

**Affiliations:** Equipment Management and UAV Engineering School, Air Force Engineering University, Xi’an 710051, China; fgq8787@163.com (G.F.); lzz144@163.com (Z.L.)

**Keywords:** object detection, YOLO, lightweight network, deep learning, surveillance footage

## Abstract

To enable person detection tasks in surveillance footage to be deployed on edge devices and their efficient performance in resource-constrained environments in real-time, a lightweight person detection model based on YOLOv8n was proposed. This model balances high accuracy with low computational cost and parameter size. First, the MSBlock module was introduced into YOLOv8n. Then, a series of modifications were made to the MSBlock structure. Next, a heterogeneous PAFPN with improved MSBlock was formed using heterogeneous convolution kernels. Finally, AKConv, a variable kernel convolution, was applied to further reduce the number of parameters and the computational cost while improving accuracy. A series of experiments demonstrated that, due to these improvements, the proposed lightweight model achieved a reduction of nearly 10% in parameter size and 5% in the floating-point computational cost compared to the original YOLOv8n. Additionally, on a custom surveillance dataset, the model shows a 1.4% improvement in mAP@0.5:0.95, and on a more complex subset of the PASVOC public dataset, the model achieved a 2.8% improvement in mAP@0.5 and a 1.2% improvement in mAP@0.5:0.95, proving the high accuracy and generalization ability of the improved lightweight model.

## 1. Introduction

Personnel detection is an important computer vision task that plays a crucial role in areas such as security monitoring, smart homes, crowd analysis, and autonomous driving. Specifically in the context of security monitoring, certain hazardous zones, military sites, and confidential areas often prohibit unauthorized personnel from intruding. Quickly and accurately identifying intruders allows for the timely detection of potential threats, a swift response, and the handling of possible security incidents, which helps to protect property, ensure personnel safety, and safeguard national security. With the development of video technology, cameras have gradually replaced manual patrols for monitoring various areas. However, to detect security incidents, monitoring personnel still need to view surveillance footage. When the monitored area is large, multiple cameras are typically installed, placing significant workload pressure on backend monitoring staff. This can lead to visual fatigue, creating security risks.

In recent years, with the continuous development of deep learning and artificial intelligence technologies, various object detection algorithms have shown remarkable performance. The application of deep learning in personnel detection technology has enhanced the effectiveness of security monitoring, to some extent improving the level of intelligent public safety management, making security measures in various places more efficient and reliable. Currently, deep learning-based object detection algorithms can be broadly classified into two categories. One category includes two-stage detection algorithms based on region proposals, such as R-CNN [1], Fast R-CNN [2], and Faster-RCNN [3], while the other category includes one-stage regression-based detection algorithms, such as YOLO [4,5,6] and SSD [7]. Compared to the former, regression-based detection algorithms do not require a proposal generation branch and can directly regress the bounding boxes and categories of objects at multiple locations in the input image [8]. These algorithms are widely used due to their faster relative speed and high accuracy. For instance, in the agricultural field, Xu et al. [9] proposed a dragon fruit ripeness detection algorithm based on YOLOv7; in remote sensing, Li et al. [10] improved YOLOv5 and built RSI-YOLO, a model suitable for remote sensing image object detection; in the medical field, Ghose et al. [11] fine-tuned YOLOv5 for colon polyp detection. However, personnel detection tasks have strict limits for model parameters and computational loads. Since personnel detection models are often deployed on different devices in varying scenarios, using models with fewer parameters and lower computational loads can reduce resource consumption while ensuring a fast response, providing low-latency feedback in real-time detection and making them suitable for edge computing devices and resource-constrained environments. Especially in real-time monitoring with cameras, models with lower parameters and computational requirements can quickly process live video streams captured by cameras on edge devices, reducing dependency on hardware resources while maintaining efficiency, thus supporting large-scale, low-cost surveillance system deployment. However, some existing algorithms do not take this into account. For example, although the railway intrusion detection method proposed by Wu et al. [12] and the highway tunnel vehicle and personnel detection algorithm proposed by Peng et al. [13] improve detection accuracy, both increase the model’s parameter size and computational load.

Typically, there is a strong negative correlation between the size of a model and its accuracy. Simply reducing the model size can lead to a decrease in computational load and parameter count, as well as an increase in speed, but it also results in a decline in detection accuracy. Therefore, it is necessary to adopt lightweight models or modify the model structure to balance model complexity and accuracy. In this regard, lightweight networks, such as SqueezeNet [14], EfficientNet [15,16], and MobileNet [17], have made significant breakthroughs, providing direction and a reference for achieving even better results in subsequent research. This paper proposes a more lightweight personnel detection model based on the YOLOv8n model, with the following main contributions:

- The use of MSBlock, a real-time detection structure with a low parameter count and computational load, and further optimization of its structure to design a better performing MSBlock module.
- The adoption of a heterogeneous convolution kernel architecture in the improved MSBlock-based PAFPN, further enhancing the model’s ability to detect objects at different scales.
- The use of AKConv variable kernel convolutions, which further reduce both the model’s parameter count and its computational load while simultaneously improving its accuracy.

## 2. Design of a Lightweight Person Detector Based on YOLOv8n

### 2.1. YOLOv8 Model

YOLOv8, released by the Ultralytics team, provides a unified framework that can handle various computer vision tasks, such as object detection, segmentation, tracking, classification, and pose estimation. The YOLOv8 network consists of three main components: a backbone network (feature extraction), neck (feature fusion module), and head (detection head). The backbone network uses a modified version of CSPDarknet-53 [18], replacing the CSP modules with C2f modules. The C2f module incorporates gradient-splitting connections, which enhance the flow of information while maintaining a lightweight architecture. At the end of the backbone, spatial pyramid fast pooling (SPPF) is used to generate feature maps of a specified size, offering a lower computational cost compared to the original SPP structure [19].

The neck utilizes the PAN-FPN architecture to achieve multi-scale feature fusion. It builds both top-down and bottom-up network structures, enabling complementary shallow location information and deep semantic features, improving the diversity and completeness of the extracted features.

The head adopts a decoupled head structure, separating classification and detection tasks. The classification task uses binary cross-entropy loss (BCE Loss), while the detection task employs distribution focal loss (DFL) and CIOU as loss functions. The final loss function is obtained by weighted averaging the three loss components.

As an anchor-free model, YOLOv8 determines positive and negative samples in a more streamlined manner. The task-aligned assigner dynamically matches positive and negative samples, allowing YOLOv8 to make better use of training data and achieve improved detection performance.

**Figure 1:**
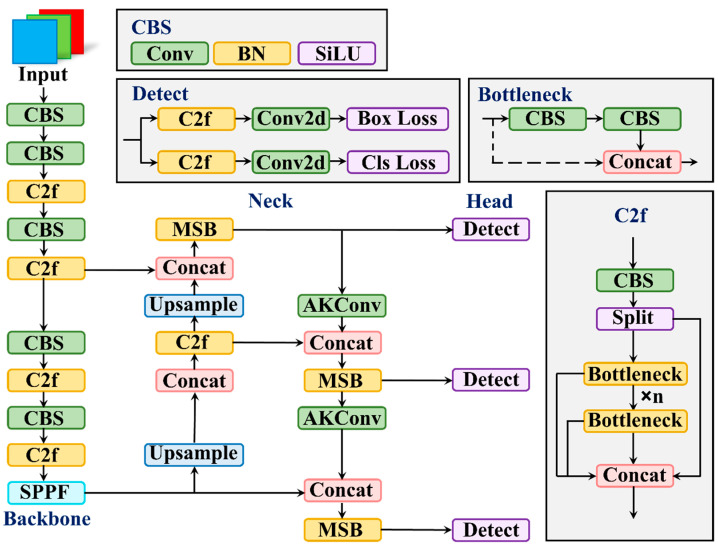
Flowchart of the improved YOLOv8n model in this paper.

In this paper, we improve upon YOLOv8’s smallest version, YOLOv8n, by incorporating AKConv and the improved MSBlock in the neck, as illustrated in Figure 1. This results in a person detector with fewer parameters and computational requirements that achieves higher detection accuracy.

### 2.2. Use of MSBlock in the YOLOv8 Model

#### 2.2.1. Structure of the MSBlock Module

**Figure 2:**
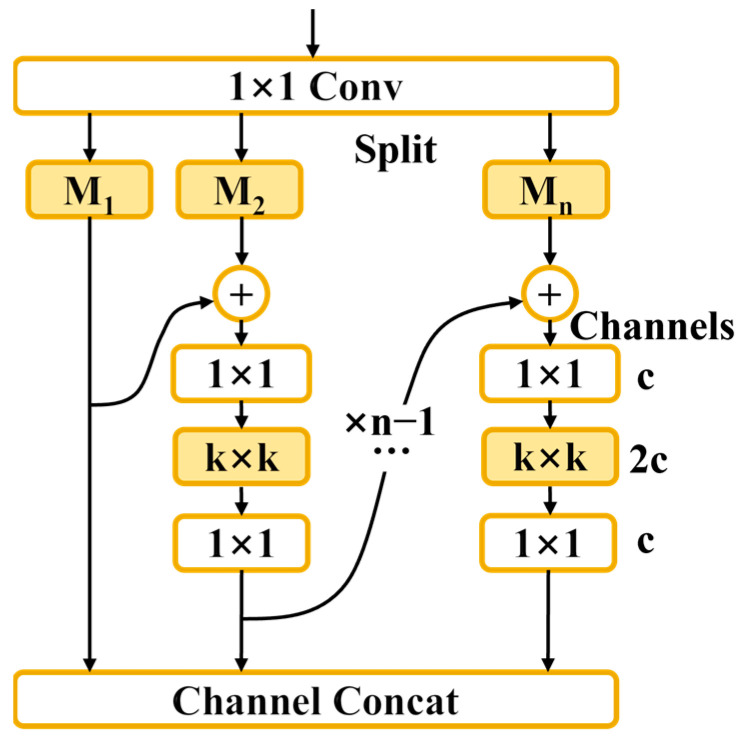
Original MSBlock structure diagram.

MSBlock, proposed by Chen et al. [20] in 2023, is a simple and effective hierarchical multi-scale feature fusion block. The structure of a single MSBlock is shown in Figure 2.

Assume the input channel number of MSBlock is inc and the output channel number is outc. In MSBlock, the number of channels in the feature map is changed from inc to outc. Then, the outc channels are evenly divided into n parts, denoted as M_1_, M_2_, ..., M_n_, with each part containing outc/n channels. The branch corresponding to M_1_ is left unchanged, while the input of the M_2_ branch is elementwise added to the output of the M_1_ branch and then sent to an inverted bottleneck block (IB). Similarly, M_3_ to M_n_ are processed in the same manner, where each subsequent branch’s input is elementwise added to the output of the previous branch before being passed into IB blocks. The IB blocks do not change the number of channels, and the output channel number of each sub-branch remains outc/n. Finally, the outputs of all the branches are concatenated to form the final feature map with outc channels. For the number of sub-branches, n is typically set to 3, as recommended in the original paper.

#### 2.2.2. Inverted Bottleneck Block

**Figure 3:**
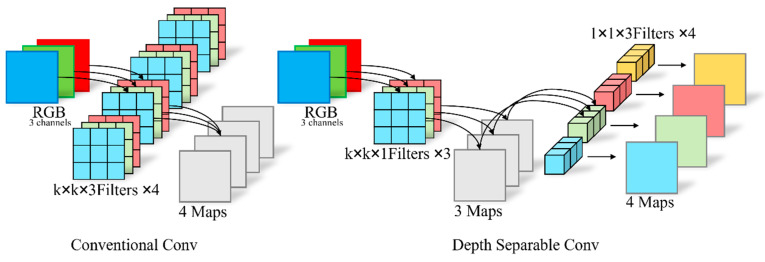
Traditional standard convolution and depthwise separable convolution architecture diagram.

The inverted bottleneck block is a feature extraction strategy with a low parameter count and computational cost. The fundamental reason for its low parameter and computational cost is the use of depthwise separable convolutions [21], as shown in Figure 3.

Depthwise separable convolution consists of two parts: depthwise convolution (DW) and pointwise convolution (PW). In depthwise convolution, separate convolution kernels, each matching the number of input channels, are applied to each channel’s feature map individually to extract spatial features from the input, resulting in a feature map with the same number of channels as the input. Pointwise convolution uses a 1 × 1 convolution to extract channel-wise features from the input, changing the dimensionality of the feature map and producing an output feature map with the specified number of channels.

Assume the kernel size of a standard conventional convolution is k × k, the number of input channels is inc, the number of output channels is outc, and the size of the output feature map is m × m. The parameter count and computational cost of the standard convolution are as follows:

(1) $$Num1_{para}\;=\;k\times k\times inc\times outc$$

(2) $$Num1_{cal}\;=\;k\times k\times inc\times outc\times m\times m$$

Assume that, in the depthwise separable convolution, the number of input channels is inc, the kernel size for depthwise convolution is k × k, the kernel size for pointwise convolution is 1 × 1, and the size of the output feature map is m × m, with outc channels. The parameter count and computational cost for the depthwise separable convolution are as follows:

(3) $$Num2_{para}\;=\;k\times k\times 1\times inc+1\times 1\times inc\times outc\;=\;k\times k\times inc+inc\times outc$$

(4) $$Num2_{cal}\;=\;k\times k\times 1\times inc\times m\times m+1\times 1\times inc\times outc\times m\times m\;\;=\;k\times k\times inc\times m\times m+inc\times outc\times m\times m$$

By comparing the parameter count and computational cost of the standard conventional convolution and the depthwise separable convolution, we obtain the ratio:

(5) $$R_{para}\;=\;\frac{Num2_{para}}{Num1_{para}}\;=\;\frac{k\times k\times inc+inc\times outc}{k\times k\times inc\times outc}\;=\;\frac{1}{outc}+\frac{1}{k^{2}}$$

(6) $$R_{cal}\;=\;\frac{Num2_{para}}{Num1_{para}}\;\;=\;\frac{k\times k\times inc\times m\times m+inc\times outc\times m\times m}{k\times k\times inc\times outc\times m\times m}\;=\;\frac{1}{outc}+\frac{1}{k^{2}}$$

In general, outc is a relatively large number, and 1/outc can be ignored. Therefore, when k is 3, 5, and 7, the parameter count and computational cost of depthwise separable convolution are approximately 1/9, 1/25, and 1/49 of the standard convolution, respectively. This demonstrates that the depthwise separable convolution has a more significant lightweight effect with larger kernel sizes.

### 2.3. Improvements to the MSBlock Structure

**Figure 4:**
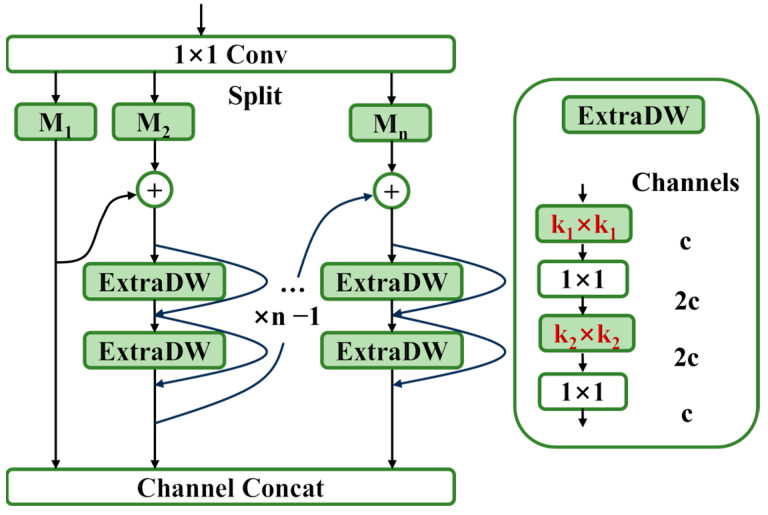
Improved MSBlock architecture diagram.

In this paper, certain improvements have been made to the MSBlock structure, and the updated MSBlock structure is shown in Figure 4.

#### 2.3.1. Replacement of Sub-Branch Modules

**Figure 5:**
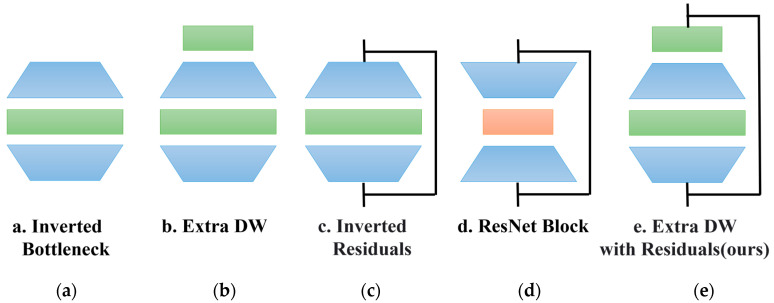
Examples of various module architectures. (**a**) Inverted bottleneck; (**b**) ExtraDW; (**c**) inverted residuals; (**d**) ResNet block; (**e**) ExtraDW with residuals (ours).

In the original MSBlock, each branch except the first one uses an inverted bottleneck (IB) structure, which applies a dimensionality expansion operation to the feature map before the depthwise separable convolution, as shown in Figure 5a. This allows depthwise convolutions to extract richer features in a higher-dimensional space. In this paper, the IB structure is replaced with an extra depthwise IB (ExtraDW) structure from the universal inverted bottleneck (UIB), as shown in Figure 5b. This structure was first proposed in 2024 in MobileNetV4 [22], and it is quite simple: an additional pointwise convolution is added before the IB, transforming the structure into a PW-DW-PW-DW form. This modification can further increase the network depth and receptive field at a lower cost.

In depthwise separable convolutions, the learning of spatial features primarily occurs in the pointwise (PW) convolution. In MSBlock, it is recommended that each branch contains 3 IBs, resulting in 3 pointwise convolutions per branch. Since each ExtraDW structure contains 2 pointwise convolutions, using only 2 ExtraDW structures per branch can achieve 4 pointwise convolutions, improving the ability to capture spatial information with fewer parameters and computational costs. Additionally, in MobileNetV4, there are two configurations for the kernel sizes of the two depthwise convolutions in ExtraDW: one is k_1_ = 3 and k_2_ = 5, and the other is k_1_ = k_2_ = k, but experimental results show that the model performs best when the two kernel sizes are the same, namely k_1_ = k_2_ = k.

#### 2.3.2. Addition of Residual Connections

The concept of residual connections was first proposed in 2016 [23] to address the problem of model performance degradation as the depth of the model increases. MobileNetV2 [24] incorporated residual connections at both ends of the inverted bottleneck block, forming an inverted residuals structure, as shown in Figure 5c. Unlike the residual structure proposed in ResNet, as Figure 5d. where the process is to first reduce the dimensionality, then apply convolution, and finally restore the dimensionality, the inverted residuals structure first increases the dimensionality, applies convolution, and then reduces the dimensionality. Inspired by the inverted residuals structure, this paper adds residual connections at both sides of each ExtraDW in the sub-branches, as Figure 5e, enabling the model to not only alleviate the vanishing gradient problem, retain original information, and enhance network depth like residual structures, but also maintain the advantages of the low parameter counts and computational costs of depthwise separable convolutions. During inference, data flow can bypass certain computational paths, directly flowing from shallower layers to deeper layers, thereby improving inference speed.

### 2.4. New Heterogeneous Convolution Kernel Selection Architecture

In the YOLO-MS model, the DW convolution kernel sizes of MSBlock used in the backbone network are set to 3, 5, 7, and 9 as the network deepens to accommodate the model’s need to extract features of different fine granularities for multi-scale detection. However, the DW convolution kernel size used in the four MSBlocks in the neck is uniformly set to 3. Experimentally, it was found that using MSBlocks in the backbone network significantly reduced the detection accuracy of the model.

For the neck, after the first C2f layer of the information flow, there is no connection to the detection head, but continued feature extraction and fusion take place, with the output features undergoing extensive subsequent processing. However, the large-range additive fusion in the MSBlock is not conducive to long-range dependencies, and it may lead to information loss at the end of the information flow. Therefore, this paper keeps the first C2f module in the neck and only replaces the subsequent three C2f modules.

**Figure 6:**
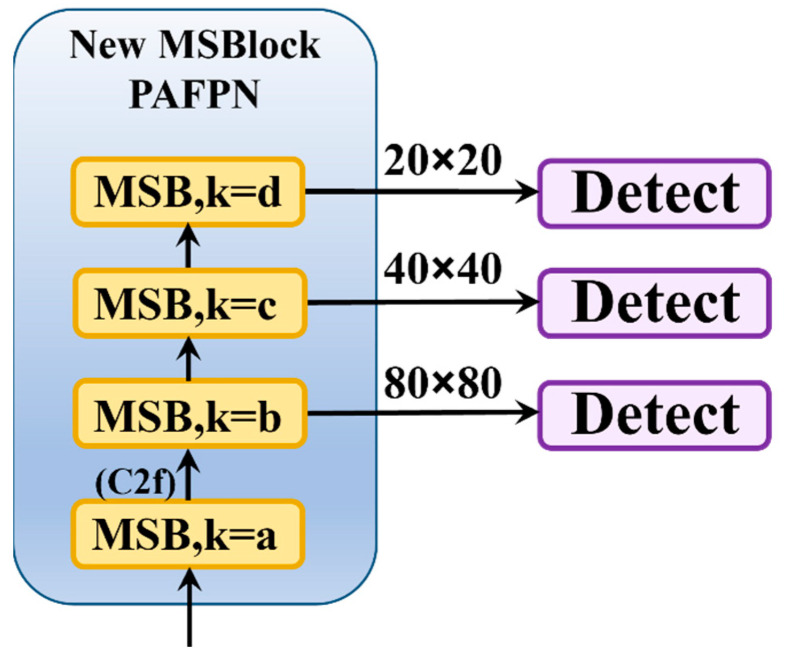
New heterogeneous convolution kernel selection architecture diagram.

The three detection heads in the YOLO series models are used to detect large, medium, and small objects, respectively. In the improved MSBlock with three detection heads at different scales, the size of the convolution kernels can alter the receptive field of each pixel in the input feature maps of the detection heads, as Figure 6. Larger convolution kernels have a wider receptive field, which helps detect larger objects, while smaller kernels are better at capturing details and smaller objects. Therefore, setting the size of the DW convolution kernels (k) to 7, 5, and 3 in the heterogeneous architecture of the improved MSBlock, connecting the large-, medium-, and small-scale detection heads, is more beneficial for the model to detect targets at different scales.

### 2.5. Use of AKconv in the Neck

**Figure 7:**
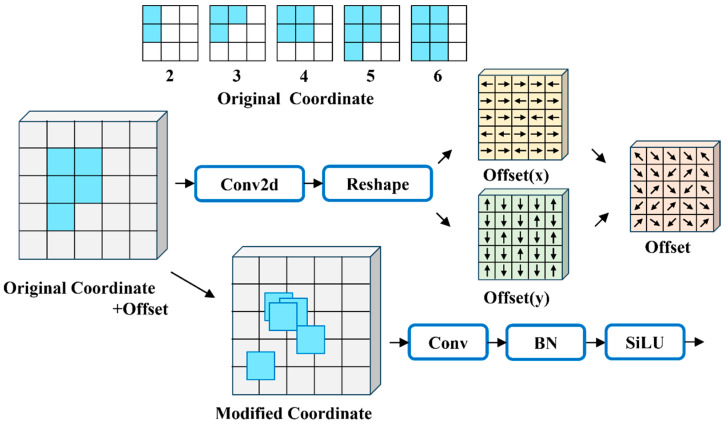
AKConv structure diagram.

AKconv [25] is a deformable convolution with variable kernels, which allows for flexible selection of the number of parameters and sampling shapes of the convolution kernels. This provides a richer set of trade-offs between network overhead and performance. The introduction of offsets further fine-tunes the sampling shape, enabling better adaptation to target variations. The structure diagram of AKConv is shown in Figure 7. In this paper, a neatly arranged original coordinate shape is used; after performing convolution on the input features, the shape is adjusted to obtain the offset. The offset is then added to the initial coordinates to obtain the final coordinates for performing convolution operations on the feature map. Since the final coordinates obtained after adjustment are usually not integers, bilinear interpolation is used to derive the corresponding values at the sampling positions on the convolved feature map.

## 3. Experiment-Related Preparation

### 3.1. Experimental Equipment and Hyperparameters

The equipment and some of the hyperparameters used in the experiments are listed in Table 1.

### 3.2. Dataset

In this paper, we use a self-constructed dataset consisting of frames extracted from numerous real surveillance videos. The dataset mainly includes nighttime surveillance footage with weak lighting conditions. The angles at which the footage is captured also vary. Additionally, the dataset includes a wide range of human poses, including standing, walking, cycling, climbing, etc. To improve the model’s performance and generalization ability and to avoid overfitting, we apply various data augmentation techniques to the images in the dataset, such as random horizontal and vertical flipping; center cropping with size transformations; and adjustments to brightness, contrast, and saturation. Since complex situations, like overlapping people, are rare in the surveillance footage, we also create a separate dataset by extracting 1000 more complex images from the “Person” category of the PASCAL VOC dataset. This is done to verify the generalization ability of the proposed method and its capability in handling more complex scenarios. In both of these datasets, the training and validation sets are split at a ratio of 8:2.

### 3.3. Evaluation Metrics

The evaluation metrics used in this paper to measure detection performance are precision, recall, F1 score, and average precision (AP). Precision refers to the proportion of true positive predictions among all predicted positive samples; recall indicates the proportion of true positive samples that were correctly predicted; the F1 score considers both precision and recall, providing a comprehensive measure of classifier performance; and average precision (AP) is calculated as the area under the PR curve. Since there is only one detection target in this paper, the mAP (mean average precision) for each class is equivalent to AP. The specific formulas for the above evaluation metrics are as follows:

(7) $$Precision\;=\;\frac{TP}{TP+FP}\;,\;Recall\;=\;\frac{TP}{TP+FN}$$

(8) $$F1_{score}\;=\;\frac{2\times precision\times recall}{precision+recall}\;=\;\frac{2TP}{2TP+FP+FN}$$

(9) $$mAP=\frac{AP}{N}=\frac{\int_{0}^{1}p\left(r\right)dr}{N}$$

## 4. Experiments and Discussion

### 4.1. MSBlock Improvement Experiments

**Figure 8:**
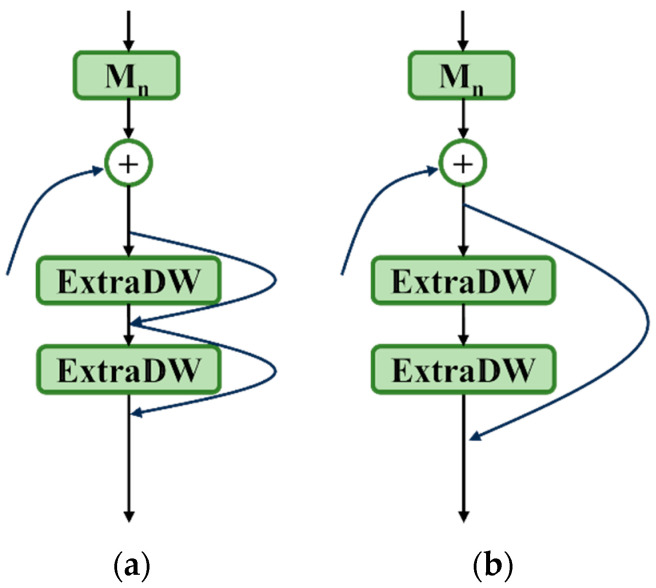
Different residual connection methods. (**a**) Small-span residual connection; (**b**) large-span residual connection.

In this paper, the improvements made to the MSBlock structure include:

- Replacing the original inverted bottleneck block structure in the sub-branches with ExtraDW;
- Setting both convolution kernels in each ExtraDW within the sub-branch to the same size and varying them according to the heterogeneous convolution kernel architecture;
- Adding small-span residual connections at both ends of each ExtraDW in the sub-branch, as shown in Figure 8a,b.

**Figure 9:**
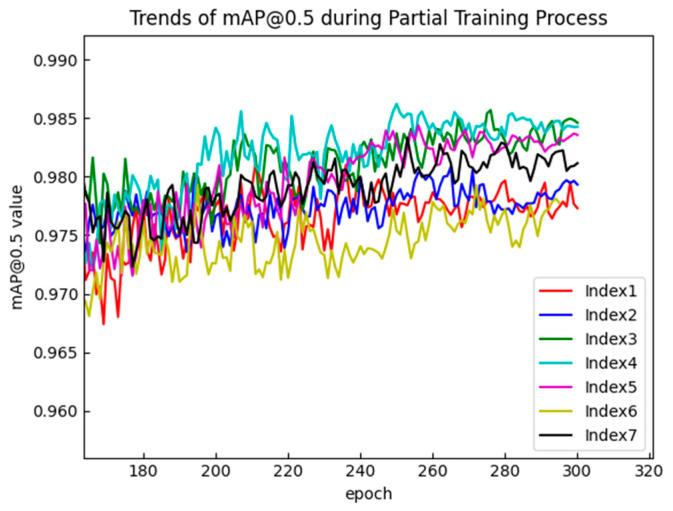
Trends in mAP@0.5 during the partial training process with different improvement conditions.

**Figure 10:**
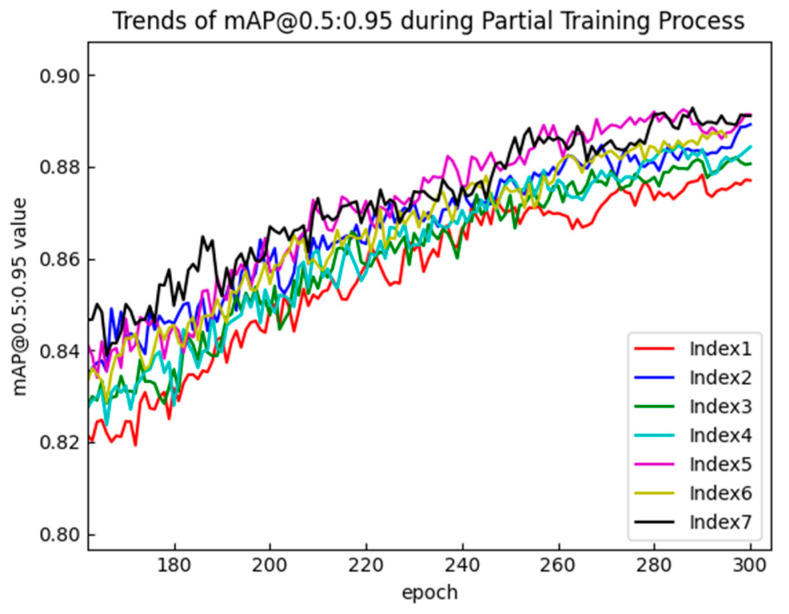
Trends in mAP@0.5:0.95 during the partial training process with different improvement conditions.

To verify the effectiveness of these improvements, a series of comparative experiments were conducted. The comparative experimental results of the three improvements in the MSBlock are shown in Figure 9 and Figure 10 and in Table 2 and the specific analysis is as follows:

- Sub-branch structure: When there is no residual connection, the accuracy of ExtraDW (index 3) in the MSblock was significantly better than that of IB (index 1). When residual connections were present, adding residual connections at both ends of ExtraDW (index 5) resulted in better accuracy compared to the inverted residual structure (index 2). Additionally, using two ExtraDW blocks in the sub-branch reduced both the number of parameters and the computational cost compared to using three IB blocks.
- Residual connections: From the comparison of the experimental results between index 4 and index 7, it can be seen that, after adding residual connections at both ends of ExtraDW, the mAP@0.5:0.95 saw a significant improvement of 1%. Experiments showed that adding residual connections on top of either IB or ExtraDW could help improve the model’s inference speed. Among them, the inference time of index 2 was reduced by 0.6ms compared to index 1, and the inference time of index 7 was reduced by 1.6ms compared to index 4. In addition, the experiment explored and compared the effectiveness of two methods: (a) connecting two small-span residuals before and after each ExtraDW, and (b) connecting large-span residuals before and after the two ExtraDW blocks in the sub-branch. Based on the experimental results (indices 6 and 7), it can be concluded that small-span residual connections are more effective in improving the model’s detection accuracy, especially with a significant improvement in the mAP@0.5:0.95 value.
- Kernel sizes in ExtraDW: For a single ExtraDW structure, choosing two DW convolution kernels with sizes k_1_ = k_2_ = k resulted in a slight increase in both the parameter count and computational cost compared to the case k_1_ = 3 and k_2_ = 5. When there was no residual connection, as shown by the experimental results in Table 2 (comparison between index 3 and index 4), the detection accuracy of both configurations was quite similar. However, the mAP@0.5:0.95 value in Table 2 corresponds to the mAP@0.5:0.95 at the point where mAP@0.5 is maximized. From Figure 10, it is evident that the mAP@0.5:0.95 curve for index 4 (k_1_ = k_2_ = k) is almost always positioned above that of index 3, and the model with k_1_ = k_2_ = k converges faster during training. Therefore, the model with k_1_ = k_2_ = k performed better. After adding residual connections, as shown by the experimental results in Table 2 (comparison between index 5 and index 7), the accuracy, especially the mAP@0.5:0.95 value, improved by 1.3% with k_1_ = k_2_ = k compared to k_1_ = 3 and k_2_ = 5.

### 4.2. AKConv Kernel Parameter Experiments

AKConv is a variable kernel convolution, and the choice of kernel size has an impact on the model’s parameters, computational cost, and accuracy. Specifically, smaller kernels help reduce the number of parameters and computational cost, but may underperform in capturing spatial features. Larger kernels can capture more spatial information but increase both the parameter count and computational cost. In the experiment, different kernel sizes (from n = 2 to n = 6) were set and compared to analyze the impact of kernel size on model performance. Based on the experimental results shown in Table 3, it was found that when n = 3, the model had relatively smaller parameters and computational costs while achieving the highest accuracy, leading to optimal overall performance.

In addition, we conducted an ablation experiment on AKConv by adding it separately to YOLOv8n. The experimental results shown in Table 4 demonstrate the effectiveness of the AKConv addition.

### 4.3. New Heterogeneous Convolution Kernel Architecture Experiments

In this section, experiments were conducted to compare different heterogeneous convolution kernel architectures.The experimental results are shown in Table 5.

Based on the experimental results, it can be concluded that, for the improved MSBlock with depthwise convolution kernels connecting the detection heads used for small-, medium-, and large-scale detection, the model with a heterogeneous architecture that used kernel sizes of 7, 5, and 3 had a lower relative parameter count and computational cost while achieving the highest accuracy.

### 4.4. Comparative Experiments with Other Models

**Figure 11:**
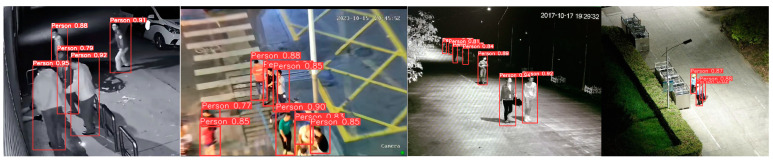
Partial detection results of the surveillance footage by the algorithm in this paper.

Based on the experimental results in Table 6, for personnel detection in surveillance footage, the lightweight model proposed in this paper reduces the parameter count by nearly 0.3 M (approximately 10%) and the computational cost by 0.5 G (approximately 5%) compared to YOLOv8n, while achieving a 1.4% improvement in mAP@0.5:0.95, resulting in higher accuracy. Additionally, compared to models with similar computational and parameter scales, such as YOLOv3n, YOLOv5n, and YOLOv6n, the proposed model shows a significant accuracy advantage. Although its accuracy is slightly lower than that of some larger models, like YOLOv8s and YOLOv9c, overall, the proposed model strikes a good balance between low computational cost and parameter size and relatively high accuracy, offering better overall performance. The partial detection results of our model are shown in Figure 11.

For devices like monitors, which are mobile or embedded systems, memory bandwidth is a critical bottleneck. By reducing the number of model parameters, less parameter data needs to be loaded, which decreases the demand for data retrieval from memory, thereby improving data loading and computation speed. The parameter file size of YOLOv8n is 5.96 MB, while the improved model’s parameter file size is 5.50 MB, a reduction of 7.7% that helps improve the real-time performance of detection. In addition, reducing the computational load helps to decrease the power consumption of the deployed device, thereby enhancing the device’s long-term stability.

### 4.5. Generalization Experiments of the Model Under Complex Conditions

**Figure 12:**
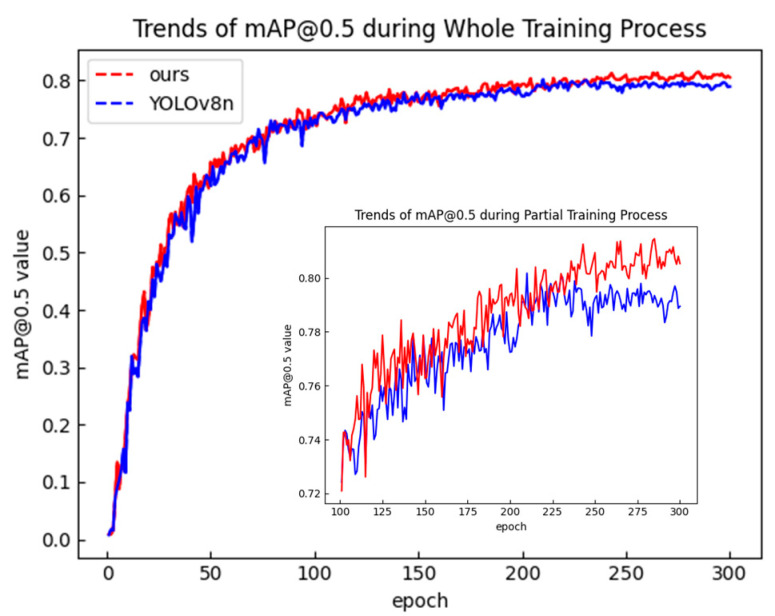
Trends in mAP@0.5 during the whole training process of our model and YOLOv8n in complex scenarios.

**Figure 13:**
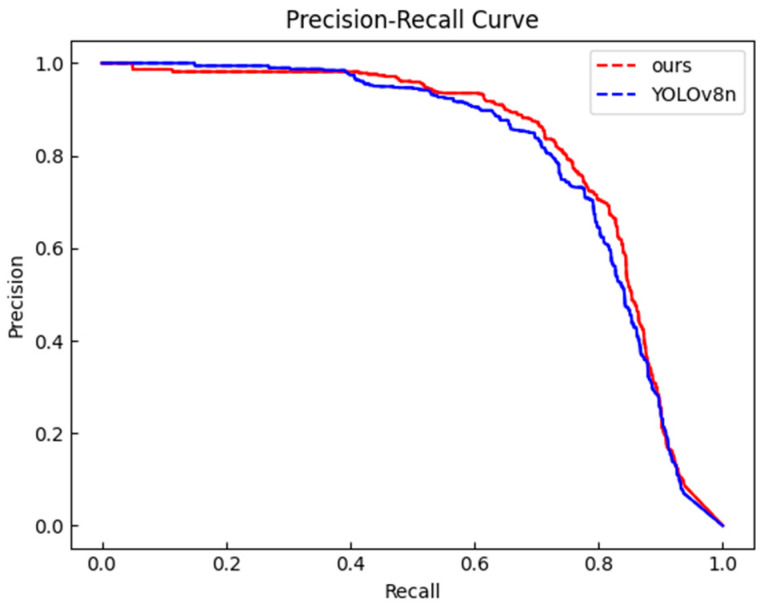
Precision–recall curve of our model and YOLOv8n in complex scenarios.

**Figure 14:**
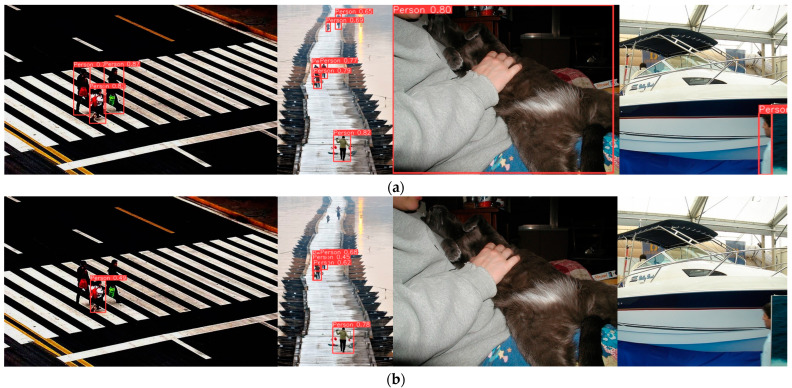
Comparison of the detection performance of our model and the original YOLOv8n in complex scenarios. (**a**) Our model; (**b**) YOLOv8n.

To evaluate the performance of the proposed algorithm for person detection in complex scenarios, experiments were conducted on a dataset consisting of 1000 randomly selected images from the “Person” category in the PASVOC dataset. The experimental results in Figure 12 and Figure 13 and Table 7 below show that the proposed algorithm improved both precision and recall, which increased the mAP@0.5 by 1.2% and mAP@0.5:0.95 by 2.0% compared to the improved YOLOv8n model, indicating that the proposed algorithm still achieved better accuracy in more complex scenarios. The partial detection results under complex conditions of our model are shown in Figure 14.

## 5. Conclusions

Real-time personnel detection in surveillance footage has a wide range of applications. To make detection models more easily deployable on edge devices, this paper proposes a lightweight personnel detection model for surveillance footage based on YOLOv8n. First, the MSBlock was introduced in the neck of the model; however, simply adding it led to a decline in detection accuracy. Then, by modifying the MSBlock through module replacement, adding residual connections, and selecting appropriate convolution kernels, the model’s detection accuracy was improved. Next, a heterogeneous convolution kernel architecture was applied to the MSBlock in the neck, enabling better adaptations to target detection at different scales. Finally, AKConv variable kernels were introduced, and the most suitable kernel parameters were selected through experiments to further enhance both the lightweight nature and detection accuracy of the model.

Through a series of improvements, the parameter count of the improved model was reduced by 10% compared to the original YOLOv8n, and the computational cost was reduced by 5%. The detection accuracy for surveillance scene detection was improved by 1.4% for mAP@0.5:0.95. Additionally, the improved model also showed a 2.8% increase in mAP@0.5 and a 1.4% increase in mAP@0.5:0.95 in more complex scenarios. Extensive experimental data indicate that the improved model struck a balance between lightweight design and high accuracy, with excellent generalization abilities and strong performance when handling complex scenarios.

## Figures and Tables

**Table 1:** The equipment and some of the hyperparameters used during the experiments.

Device	Parameter	Hyperparameter	Value
CPU	Intel(R) Core(TM) i7-14650HX	Training Strategy	SGD
RAM	32 GB	Momentum	0.937
Operating System	Windows	Initial Learning Rate	0.01
GPU	NVIDIA GeForce RTX 3060 Laptop GPU	Final Learning Rate	0.0001
GPU Memory	12 GB	Weight Decay	0.0005
Programming Tool	PyCharm 2023.3.3	Batch Size	4
Programming Language	Python	Warm-up Epochs	3
Deep Learning Framework	PyTorch 2.3.1	Total Epochs	300

**Table 2:** Performance comparison of the model with MSBlock under different improvement conditions (without adding AKConv).

Index	Improvement of MSblock			Parameters	FLOPs	mAP@0.5	mAP@0.5:0.95
	Sub-Branch Structure	Residual	DW Convolution Kernel Size [k_1_;k_2_][a;b;c;d]				
1	IB	No	[k][-;3;5;7]	2.870 M	7.9 G	98.1%	85.3%
2	IB	Yes(a)	[k][-;3;5;7]	2.870 M	7.9 G	98.1%	88.0%
3	ExtraDW	No	[3;k][-;3;5;7]	2.813 M	7.8 G	98.6%	87.8%
4	ExtraDW	No	[k;k][-;3;5;7]	2.825 M	7.8 G	98.6%	87.7%
5	ExtraDW	Yes(a)	[3;k][-;3;5;7]	2.813 M	7.8 G	98.4%	88.6%
6	ExtraDW	Yes(b)	[k;k][-;3;5;7]	2.825 M	7.8 G	97.9%	85.7%
7	ExtraDW	Yes(a)	[k;k][-;3;5;7]	2.813 M	7.8 G	98.3%	88.7%

**Table 3:** Performance comparison of different AKConv kernel sizes (with improved MSBlock).

AKConv Kernel Parameter	Parameters	FLOPs	mAP@0.5	mAP@0.5:0.95
2	2.689 M	7.6 G	98.6%	89.0%
3	2.713 M	7.6 G	98.6%	89.9%
4	2.737 M	7.7 G	98.4%	88.9%
5	2.761 M	7.7 G	98.1%	87.8%
6	2.785 M	7.7 G	98.3%	86.0%

**Table 4:** AKconv ablation experiment (without improved MSBlock).

Model	Parameters	FLOPs	mAP@0.5	mAP@0.5:0.95
YOLOv8n	3.006 M	8.1 G	98.4%	88.5%
YOLOv8n + AK	2.894 M	7.9 G	98.6%	88.6%

**Table 5:** Performance comparison of the model under different heterogeneous convolution kernel architectures.

Architecture [a;b;c;d]	Parameters	FLOPs	mAP@0.5	mAP@0.5:0.95
[3;3;3;3]	2.636 M	7.5 G	98.2%	87.5%
[-;3;3;3]	2.676 M	7.6 G	98.2%	86.5%
[-;5;5;5]	2.697 M	7.7 G	98.2%	86.1%
[-;3;5;5]	2.694 M	7.6 G	98.7%	86.9%
[-;3;5;7]	2.713 M	7.6 G	98.6%	89.9%
[-;7;5;3]	2.689 M	7.7 G	98.6%	89.6%

**Table 6:** Performance comparison of mainstream models for surveillance footage detection.

Model	Parameters	FLOPs	mAP@0.5	mAP@0.5:0.95
YOLOv3n	4.540 M	12.0 G	97.9%	83.1%
YOLOv3-tiny	12.128 M	18.9 G	98.2%	87.6%
YOLOv5n	2.503 M	7.1 G	98.1%	85.8%
YOLOv5s	9.120 M	23.8 G	98.3%	90.0%
YOLOv6n	4.234 M	11.8 G	97.4%	84.7%
YOLOv7s	9.319 M	26.7 G	96.3%	77.1%
YOLOv8n	3.006 M	8.1 G	98.4%	88.5%
YOLOv8s	11.126 M	28.4 G	97.8%	90.9%
YOLOv9c	25.320 M	102.3 G	99.2%	92.4%
RT-DETR-n	8.011 M	14.4 G	95.2%	78.4%
RT-DETR-n(Res50)	2.503 M	7.1 G	98.1%	85.8%
Model in paper [12]	4.760 M	14.6 G	98.5%	90.5%
Ours	2.713 M	7.6 G	98.6%	89.9%

**Table 7:** Performance comparison between our model and YOLOv8n in complex scenarios.

Model	Parameters	FLOPs	Precision	Recall	mAP@0.5	mAP@0.5:0.95
YOLOv8n	3.006 M	8.1 G	83.8%	70.1%	80.2%	44.6%
Ours	2.713 M	7.6 G	84.3%	71.3%	81.4%	46.6%

## Data Availability

The data presented in this study are not available upon request from the corresponding author due to the involvement of personal privacy in some of the monitoring footage.
